# Effect of the Addition of Amine in Organophosphorus Compounds on Molecular Structuration of Ionic Liquids–Application to Solvent Extraction

**DOI:** 10.3390/molecules25112584

**Published:** 2020-06-02

**Authors:** Soumaya Gmar, Fabrice Mutelet, Alexandre Chagnes

**Affiliations:** 1 Université de Lorraine, CNRS, GeoRessources, F-54000 Nancy, France; soumaya.gmar@univ-lorraine.fr; 2 Laboratoire Réactions et Génie des Procédés, Université de Lorraine, 1 rue Grandville, BP 20451 Nancy, France; fabrice.mutelet@univ-lorraine.fr

**Keywords:** viscosity, molar volume, heat of mixture, solvent extraction, ionic liquids

## Abstract

Variation of dynamic viscosity, density and enthalpy as a function of mole fraction of amine (tri-n-octylamine, triisooctylamine, bis(2-ethylhexyl)amine) in bis(2-ethylhexyl) phosphoric acid (D2EHPA) or Cyanex 272, (bis(2,4,4-trimethylpentyl)phosphinic acid) has been determined at 25 °C. Valuable information regarding structuration and destabilization of the corresponding ionic liquids has been deduced from these data. A simple model describing the variation of dynamic viscosity as a function of mole fraction of amine has been used to determine the speciation in these mixtures. Extraction tests of cobalt(II) and nickel(II) by D2EHPA-amine mixtures have shown the highest cobalt(II)-nickel(II) selectivity has been achieved with D2EHPA-2-ethylhexylamine mixture as cobalt(II) extraction efficiency of 77% was obtained, while no significant nickel(II) extraction was observed at a chloride concentration of 3 mol·L^−1^.

## 1. Introduction

Liquid-liquid extraction is a mature technology usually used in hydrometallurgy to recover metals from leaching solution mainly produced by acid digestion of primary and secondary resources [1]. This method relies on the contact of the aqueous leaching solution with a non-miscible organic solvent composed of an extractant, a diluent, and in some cases a phase modifier. Solvent extraction efficiency and selectivity mainly depends on the formulation of the extraction solvent. In particular, the chemical structure of extractant plays a crucial role [1,2]. Therefore, more and more studies are focused on synthesis of new extractants in order to improve the efficiency and the selectivity of solvent extraction processes [3,4]. Another approach consists in mixing commercial extractants to formulate new solvents exhibiting synergistic properties, i.e., a significant increase in extraction efficiencies towards targeted metals. In such synergistic systems, the distribution ratio obtained by mixing two extractants is greater than the sum of either extractant functioning alone. In other words, the synergistic effect can be assayed by calculating the synergistic coefficient *SC* expressed as follows [5,6]:

(1) $$SC=\frac{D_{mix}}{D_{A}+D_{B}}$$

where *D_A_* and *D_B_* are the distribution ratios of the metal ion by single extractant A and B, while *D_mix_* represents the distribution ratio by the mixture of A and B. 

Usually, synergistic effects take place when solvating and acidic compounds are mixed together because solvating molecules improves the extraction by rendering the complex more liphophilic (replacement of water molecules in the first coordination sphere of metal ion) [1]:

(2) $$M^{n+}+n\bar{HL}+x\bar{S}⇌\bar{ML_{n}S_{x}}+nH^{+}$$

In Equation (2), *M^n+^* is metal ion, *HL* is acidic extractant, *S* is organophosphorus compound and the overline bars denote the species in the organic phase.

The enhancement of the distribution ratio can be as high as 10^6^ and is attributed to the higher solubility of the synergistic adduct in the organic phase.

Synergistic effects were also reported by mixing amine and organophosphorus acid [7,8,9,10,11,12,13]. For instance, Liu et al. [10] showed synergistic extraction of rare-earth elements (REE) by mixing organophosphorus compounds such as Cyanex 272 (bis(2,4,4-trimethylpentyl)phosphinic acid), PC88A (2-ethylhexyl-2-ethylhexyl phosphonic acid) or D2EHPA (bis(2-ethylhexyl)phosphoric acid) with Alamine 336 (a blend of octyl and decyl tertiary amines). In such systems, it was observed that the synergistic coefficient (SC, Equation (1)) for the extraction of REEs by binary mixtures of 0.5 mol·L^−1^ organophosphorus acid and 0.5 mol·L^−1^ Alamine 336 depends on the nature of the extractants and the targeted metal. Indeed, SC decreased in the following order: Cyanex 272 (SC(La) = 5.5, SC(Ce) = 25.7, SC(Pr) = 105.7, and SC(Nd) = 66.2) > PC88A (SC(La) = 2.0, SC(Ce) = 6.2, SC(Pr) = 6.3, and SC(Nd) = 6.8) > D2EHPA (SC(La) = 0.86, SC(Ce) = 0.90, SC(Pr) = 1.06 and SC(Nd) = 1.08). 

Liquid-liquid extraction of metal cations by organophosphorus compounds occurs typically according to a cationic exchange equilibrium:

(3) $$M^{n+}+n\bar{HL}⇌\bar{ML_{n}}+nH^{+}$$

When introducing an amine into the organic phase containing an organophosphorus acid, strong interactions between the organophosphorus acid and the organic amine occur resulting in the formation of a stable ion pair adduct [14]:

(4) $$M^{n+}+n\bar{HL}+x\bar{R_{3}N}⇌\bar{ML_{n}\cdot mR_{3}N}+nH^{+}$$

Such a reaction may change the extraction efficiency in either a positive (synergism, SC > 0) or a negative (antagonism, SC < 0) way depending on the interactions and speciation in solution, which depends at their turn on the chemical structure of amines and organophosphorus acids.

Therefore, it is of great interest to investigate speciation and interactions in mixtures of amines and organophosphorus acids as a function of the nature of these reagents and their mole fraction. For this goal, dynamic viscosity, density and heat measurements have been carried out in mixtures of several amines (tri-2-ethylhexylamine, triisooctylamine, tri-n-octylamine triisooctylamine, tri-n-octylamine) and organosphosphorus acids (D2EHPA or Cyanex 272). For the first time, viscosity measurements have been used to determine the stoichiometry of the species as a function of the composition of amine-organophosphorus acid mixtures. The resulting extraction efficiencies of cobalt(II) by amine-D2EHPA mixtures and the selectivity towards nickel(II) have been finally determined and discussed in the light of the speciation in organic phase and chemical structures of amines.

## 2. Material and Methods

Tri-2-ethylhexylamine (TEHA, Aldrich, France, purity > 97%), triisooctylamine (TIOA, Aldrich, technical grade), tri-n-octylamine (TOA, Aldrich, France, purity = 98%), tri-n-octylamine (Aldrich, France, purity = 98%), bis(2-ethylhexyl)phosphoric acid (D2EHPA) (Aldrich, France, purity = 98%), Cyanex 272 (Solvay, Niagara Falls, Ontario, Canada) were used without any further purification. Mixtures were prepared by weighting the appropriate amount of amine and organophosphorus acid by using a Mettler Toledo MS-TS balance (4 digits). 

CoSO_4_·7H_2_O, NiCl_2_, NaCl and HCl (Aldrich, purity = 98%) were used to prepare feed solutions for liquid-liquid extraction experiments. Feed solutions of Ni(II) and Co(II) at 0.008 mol·L^−1^ were prepared by dissolving the required amount of salts in 0.1 mol·L^−1^ hydrochloric acid solutions containing various amount of sodium chloride in order to obtain aqueous solution containing total chloride concentrations varying from 1 mol·L^−1^ and 5 mol·L^−1^. 

Density and viscosity were determined using an Anton Paar DMA 4500M combined densimeter viscometer (Anton Paar, Paris, France). The sample was introduced into a U-shaped borosilicate glass tube that was being excited to vibrate at its characteristic frequency. The characteristic frequency changed depending on the density and the viscosity of the sample. The combined densimeter viscometer was calibrated with air, demineralized water, tri-n-octylamine and D2EHPA at 20 °C. The viscosity and density values of tri-n-octylamine, Cyanex 272 and D2EHPA were compared with those previously reported in the literature [15] and good agreements were found.

Calorimetric measurements were carried out in a Setaram C80 calorimeter (Setaram, Paris, France) at 25 °C and atmospheric pressure. A full description of the procedure is given in the previous work [16]. In a few words, a cylindrical reversal-mixing cell containing two compartments was used. The amine and D2EHPA or Cyanex 272 were placed in the bottom and top compartments, respectively. Temperature of the calorimeter was maintained during 3 h before measurements. The calorimeter was then turned 180° until the maximum heat flux is reached. The movable cap used to separate the compounds allowed to obtain good mixing. The apparatus uncertainty was evaluated at ±3%. 

Liquid-liquid extraction experiments were carried out by mixing 10 mL of aqueous phases and 10 mL of the aqueous of organic phases for 15 min at 150 rpm at room temperature by means of a Gerhard thermoshaker (Grhard, Strasbourg, France). Organic and aqueous phases were separated after centrifugation at 3000 rpm for 2 min with a SIGMA 2-16P centrifuge (VWR, Paris, France). Nickel and cobalt concentrations in the aqueous phases after liquid-liquid extraction were determined by measuring the absorbances of the aqueous phase at 410 nm and 511 nm, respectively, by means of a Cary 60 UV-Vis spectrometer (UV-Vis cube with a pathway length of 1 cm) (Agilent, Paris, France). Metal ion concentration transferred from the aqueous phase into the organic phase was then deduced by mass balance in order to calculate the extraction efficiencies and the distribution ratios of nickel and cobalt. Experiments were duplicated and the experimental error on the distribution coefficients of metals was estimated to be within 5%.

Table 1, Table 2 and Table 3 gathers the experimental values of viscosity, density and enthalpy for the mixtures of amine and D2EHPA or Cyanex 272 as a function of mole fraction of an amine.

## 3. Results and Discussion

### 3.1. Physicochemical Properties

**Figure 1:**
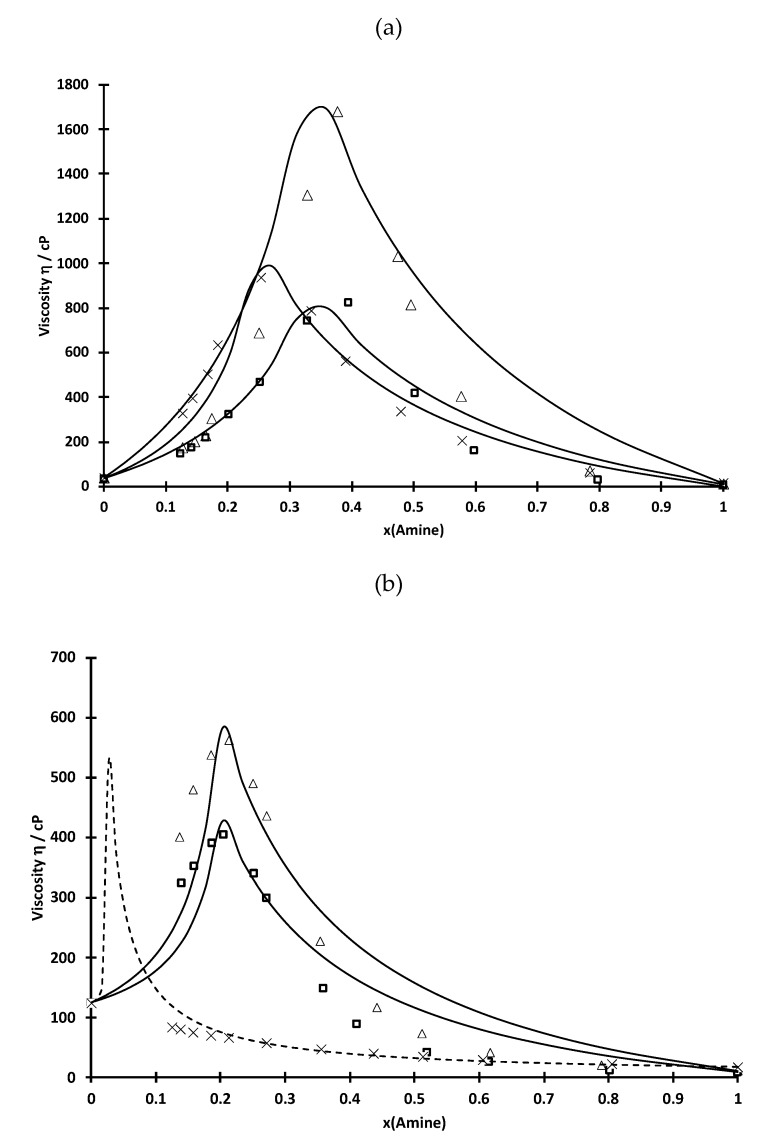
Viscosity of (**a**) D2EHPA-amine and (**b**) Cyanex 272-amine mixtures as a function of mole fraction of TOA (square), TIOA (triangle) and TEHA (cross) at 25 °C.

Figure 1 shows the variation of the dynamic viscosity of D2EHPA-amine and Cyanex 272-amine mixtures as a function of amine mole fraction at 25 °C (values are reported in Table 1 and Table 2). The dynamic viscosity increases until a threshold value (x_amine,max_), and then decreases, when amine is added into Cyanex 272 or D2EHPA. This threshold value x_amine,max_ depends on the type of amine added into the organophosphorus compound and corresponds to the formation of an ionic liquid due to the reaction between the amine and the organophosphorus acid. It is interesting to point out that no maximum of viscosity for the Cyanex 272-TEHA mixture is not observed likely because the maximum of viscosity is located at very low mole fraction of amine. In the binary mixtures, the organophosphorus acid acts as a proton donor while a primary, secondary or tertiary amine has unshared electron pair on the nitrogen atom. Therefore, an amine has the tendency to react with an acidic extractant. In general, three kinds of interaction may occur between acid and amine extractants, i.e., (i) ion-pair (R_3_NH^+^, A^−^), (ii) hydrogen bonding (R_3_NH-A) and (3) binding which has an intermediate characteristic as represented by the location of the proton (R_3_N-H-A) [17,18]. The nature of the interactions in the mixture depends on the properties of the extractants involved as well as the concentration ratio of components.

The increase in the dynamic viscosity may be explained by the reaction between the amine and D2EHPA or Cyanex 272, which may lead to the formation of solvated ion pairs by strong electrostatic interactions:

(5) $$R_{3}N+aHL\to \;\left\{R_{3}NH^{+},L^{-}\right\}{\left(HL\right)}_{a-1}$$

When x_amine_ < x_amine,max_, HL is in excess and the mixture contains HL and {R_3_NH^+^, L^−^}(HL)_a−1_. The η-values increase due to the increase in the concentration of the highly viscous species {R_3_NH^+^, L^−^}(HL)_a−1_. The maximum value of viscosity (η_max_) is, thus, reached when only {R_3_NH^+^, η-values }(HL)_a−1_ is present in the solution, i.e., when the mole fraction of the amine corresponds to the stoichiometric ratio between the amine and the HL. 

In the rich-amine region, the {R_3_NH^+^, L^−^}(HL)_a−1_ species might react with the amine to form another highly viscous species, i.e., {R_3_NH^+^, L^−^}:


{R_3_NH^+^, L^−^}(HL)_a−1_ + (a − 1)R_3_N → a{R_3_NH^+^, L^−^} (6)

However, the decrease in dynamic viscosity when the amine is added in excess into the organophosphorus acid cannot be explained by the formation of {R_3_NH^+^, L^−^} as the viscosity of this species is likely more or less the same as {R_3_NH^+^, L^−^}(HL)_a−1_. Therefore, the decrease in the dynamic viscosity for x_amine_ > x_amine,max_ (amine in excess to organophosphorus acid) is likely due to the dilution of the amine-organophosphorus species (it is not possible to give the exact nature of this species as it can be an ion pair formed between the amine and the organophosphorus acid or the other species above-mentioned, i.e., R_3_NH-A (hydrogen bonding) or (R_3_N-H-A), which is an intermediate species with proton delocalized between the amine and the conjugated base of the acidic extractant). 

Examination of the dynamic viscosity values as a function of the type of the amine in the mixtures shows that the dynamic viscosity is dependent on the chemical structure of the amine and varies as follows: TIOA >> TEHA > TOA. Branches in alkyl chains of the amines seem to hinder the flow transport the flow transport. Furthermore, the D2EHPA-amine mixtures exhibit higher viscosity values than the Cyanex 272-amine mixtures. This is in accordance with the previous studies [10,19] and with the fact that interactions between an amine and an acid extractant are proportional to the acidity of the acididic extractants (acidity of D2EHPA is greater than that of Cyanex 272 in view of pK_a_ values [20]). Liu et al. [10] estimated that the strength of the interactions between D2EHPA and Alamine 336 (mainly composed of tri-n-octylamine) is about three times as strong as Cyanex 272 and Alamine 336 by calculating excess values of dielectric constants. 

Molar volumes at 25 °C of the D2EHPA-amine and Cyanex 272-amine mixtures have been calculated from density values reported in Table 1 and Table 2. The difference of molar volume at 25 °C (*V_m_*^≠^) was calculated by using the following equation:

(7) $$V_{m}^{\neq }=\frac{\left(x_{1}M_{1}+x_{2}M_{2}\right)}{\rho }-\left(\frac{x_{1}M_{1}}{\rho_{1}}+\frac{x_{2}M_{2}}{\rho_{2}}\right)$$

where ρ is the density of the solution, ρ_1_ and ρ_2_ are the densities of an amine (TOA, TIOA, TEHA) and an organophosphorus acid (D2EHPA or Cyanex 272), respectively. *M*_1_ and *M*_2_ are molecular weights of an amine and an organophosphorus acid, respectively. It is interesting to notice that excess volume molar cannot be defined in this case because of the reaction between amine and organophosphorus extractants (excess molar functions can be defined only providing that there is no chemical reaction during mixing).

**Figure 2:**
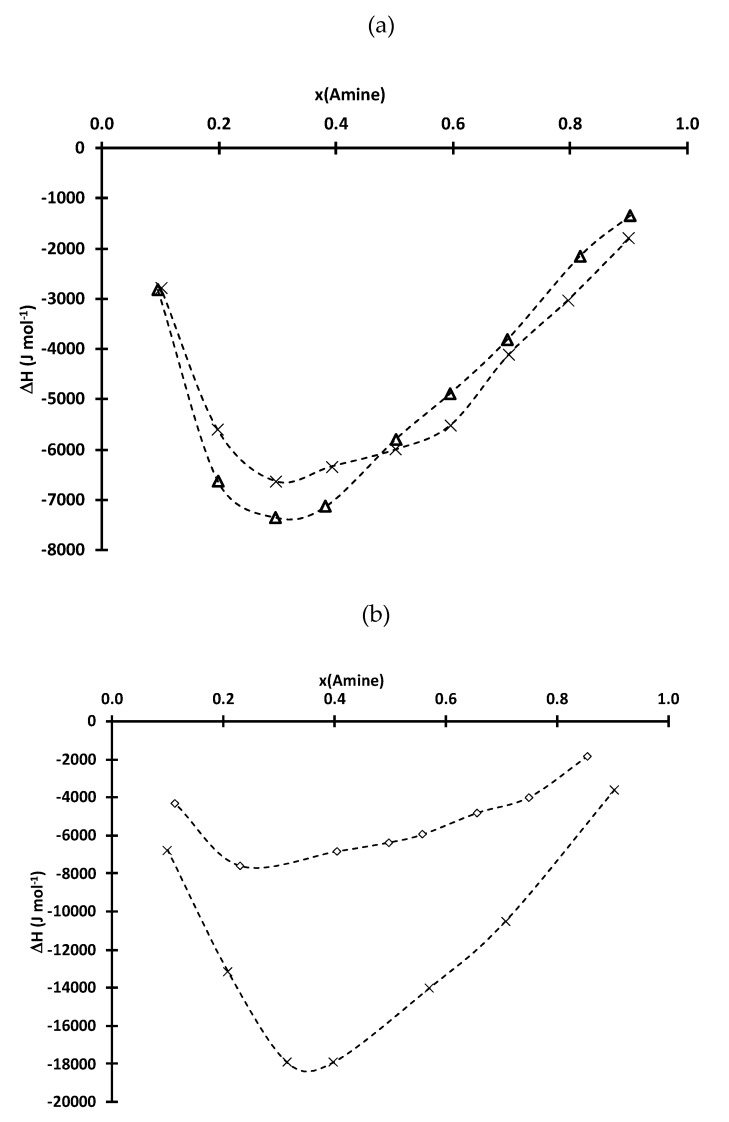
Global enthalpy during mixing of (**a**) Cyanex 272 and (**b**) D2EHPA with TIOA (cross), TOA and TEHA (diamond) as a function of mole fraction of the amine at 25 °C.

The variations of *V*_*m*_^≠^ as a function of the mole fraction of an amine in the D2EHPA-amine and Cyanex 272-amine mixtures were fitted by using the following Redlich-Kister equation (Figure 2) [21]:

(8) $$V_{m}^{\neq }=x_{1}\left(1-x_{1}\right)\overset{n}{\underset{i=0}{\sum }}A_{i}{\left(2x_{1}-1\right)}^{i}$$

where *x*_1_ represents the mole fraction of an amine in the D2EHPA and Cyanex 272-amine mixtures. The values of the adjustable parameters *A_i_* in Equation (8) are gathered in Table 3.

*V_m_*^≠^ gives information on ion pair formation [Equations (5) and (6)] and interaction changes when an amine is added into Cyanex 272 or D2EHPA. In the D2EHPA-amine mixtures, there is no real influence of the chemical structure of the amine on the strength of the electrostatic interactions (and therefore the stability of the ion-pairs formed between D2EHPA and an amine) since *V_m_*^≠^ values are quite similar for the D2EHPA-TOA, D2EHPA-TIOA and D2EHPA-THEA mixtures. 

More important differences in *V_m_*^≠^ values are observed for the Cyanex 272-amine mixtures as *V_m_*^≠^ values in the presence of TEHA mixtures are lower than those obtained for Cyanex 272-TIOA and Cyanex 272-TOA mixtures. Branching of amine seems to destabilize Cyanex 272-amine ion pairs. 

Furthermore, it is interesting to highlight that the minimum of *V_m_*^≠^ is located at the same mole fraction as the maximum of viscosity for all investigated systems. This is not really surprising as a minimum of *V_m_*^≠^ denotes the presence of strong interactions in the solution such as electrostatic interactions arising from ion-pairs formation of {R_3_NH^+^, L^−^}(HL)_a−1_. 

**Figure 3:**
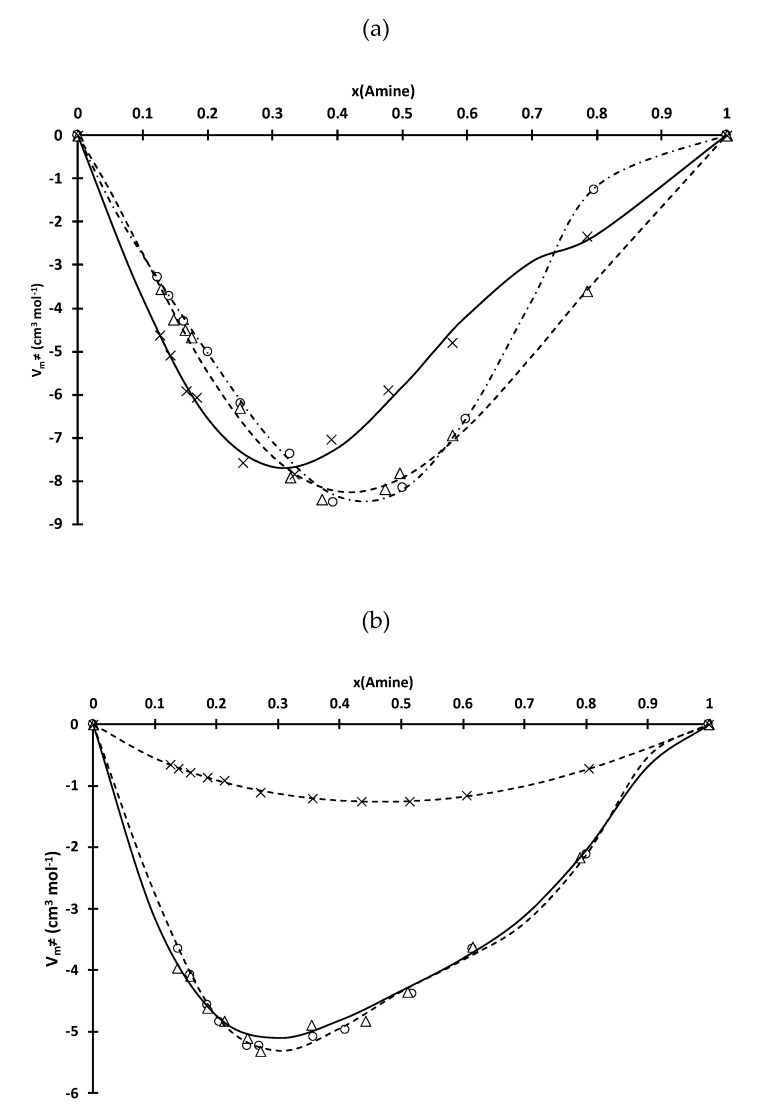
Difference in molar volume (*V_m_*^≠^, see Equation 7) at 25 °C as a function of mole fraction of the amine for mixtures of (**a**) D2EHPA or (**b**) Cyanex 272 and TOA (circle), TIOA (triangle) and TEHA (cross). Experimental points were fitted with Redlich-Kister equation (see Equation (8) and Table 4).

Calorimetric measurements reported in Figure 3 were performed to study the influence of mole fraction of an amine on the global enthalpy expressed as:

(9) $$∆H=H^{mix}+\Delta_{r}H$$

where **H^mix^** and ∆*_r_H* denote the enthalpy of mixture and the enthalpy of the reaction reported in Equation (5), respectively, when an amine is added into D2EHPA or Cyanex 272.

It is not possible to discriminate *H^mix^* and ∆*_r_H*. However, *H^mix^* is likely negative since strong attractive forces between the mixed molecules usually result in a release of heat. Likewise, acid-base reactions usually exhibit negative values. As a consequence, the global enthalpy of a mixture is negative. The minima of the global enthalpies are located at the same mole fractions of amine as those observed for η and *V_m_*^≠^ (Figure 1 and Figure 2).

There is no significant difference in global enthalpies for Cyanex 272-TIOA and Cyanex 272-TOA likely because there is no significant difference in enthalpy of the reaction between these systems, and because the electrostatic interactions are of the same order of magnitude as it was confirmed by comparing *V_m_*^≠^ values (see above). Conversely, the global enthalpies obtained for the D2EHPA-TIOA mixtures are drastically greater than those obtained for the D2EHPA-TEHA mixtures. This difference can be explained by the existence of a very exothermic reaction between D2EHPA and TEHA since the analysis of *V_m_*^≠^ in Figure 3 showed the magnitude of the electrostatic interactions between D2EHPA and TIOA or TEHA are similar.

The variation of the viscosity (η) in the D2EHPA-amine or Cyanex 272-amine mixtures as a function of mole fraction of amine (x_1_) can be expressed by using the following equation:

η = x_1_η_1_ + (1 − x_1_)η_2_ + x_IP_η_IP_ + η^EX^ (10)

where η_1_, η_2_, η_IP_ and η^EX^ denote the viscosity of the pure amine, the pure organophosphorus acid (D2EHPA or Cyanex 272), the ion pair {R_3_NH^+^, L^−^}(HL)_a−1_ as well as the excess of viscosity due to interaction in the solution (non-ideality).

**Figure 4:**
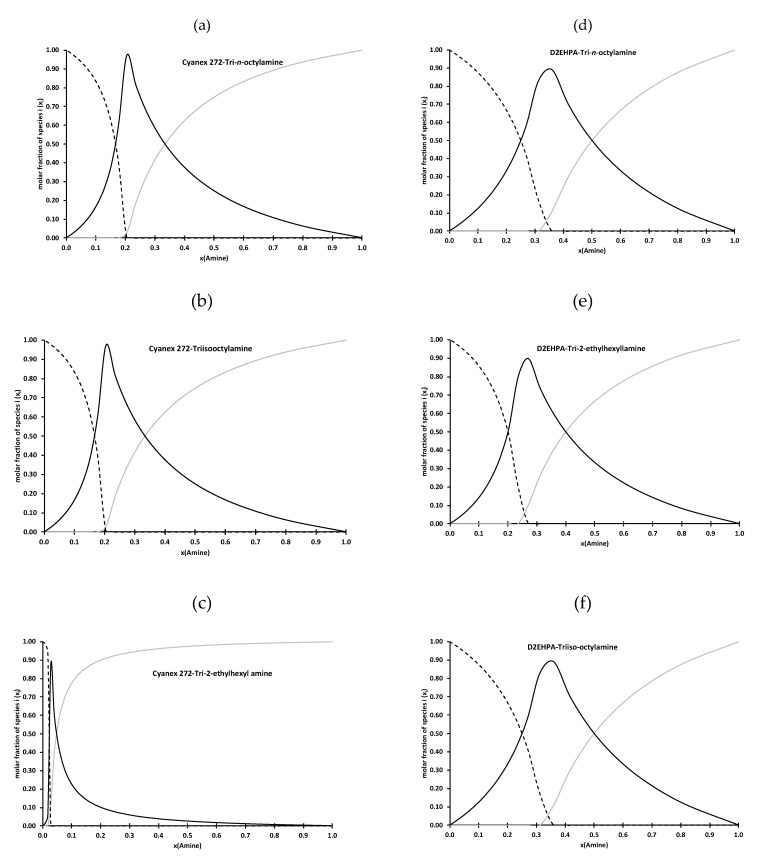
Speciation diagrams at 25 °C in mixtures of Cyanex 272 (**a**–**c**) or D2EHPA (**d**–**f**) and TOA, TIOA and TEHA as a function of mole fraction of the amine (gray curve = free amine, dotted line = free organophosphorus acid, black line = R_3_NHL(HL)_a−1_ species).

It is therefore possible to calculate the variation of the viscosity as a function of the mole fraction of amine by considering, in a first approximation, that the system is ideal (Equation (10) with η^EX^ = 0). The straight lines in Figure 1 represent the results of the calculations with the most appropriate values of η_IP_ (estimated value of the dynamic viscosity of {R_3_NH^+^, L^−^}(HL)_a−1_) and the most appropriate values of the stoichiometric ratio (a) (see Table 5). The corresponding chemical formulae of each ion-pairs expected to be formed in the Cyanex 272- and D2EHPA-amine mixtures are reported in Table 5. From these results, it was also possible to deduce the speciation in these mixtures as a function of the mole fraction of the amine (Figure 4).

The maximum values of viscosity calculated by this simple model are in a good agreement with the experimental data. Obviously, there are discrepancies between the calculated and the experimental values of η for rich and poor-amine mixtures as the model considers an ideal mixture. Values of η_IP_ reported in Table 5 show that the electrostatic interactions between D2EHPA and an amine are stronger than those between Cyanex 272 and an amine in accordance with the study of molar volumes (see above). 

### 3.2. Effect of Amine Molar Fraction on the Liquid-Liquid Extraction Properties of the Ionic Liquids

Performances in liquid-liquid extraction depend mainly on extractant-extractant and metal-extractant interactions in the absence of diluent like in the present case. The preceding results mentioned above have shown that the strength of the interactions between D2EHPA and an amine are stronger than between Cyanex 272 and an amine. In this part, the influence of an amine in the D2EHPA-amine mixtures on cobalt(II) extraction from acidic chloride media has been investigated because it is expected that the strong interactions between D2EHPA and an amine will favor the formation of {R_3_NH^+^, L^−^}(HL)_a−1_. In particular, the influence of chloride concentration and the nature of an amine on cobalt(II) extraction and cobalt(II)-nickel(II) separation have been investigated.

**Figure 5:**
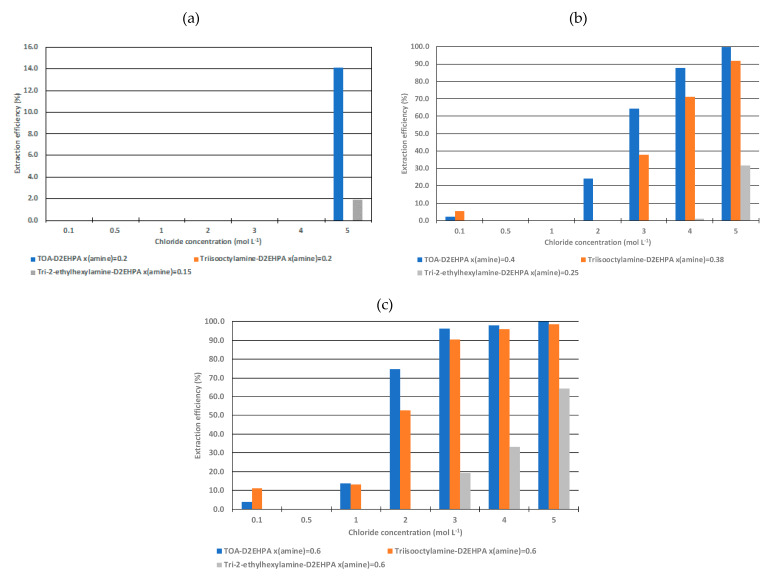
Extraction efficiency of cobalt vs. initial chloride concentration by the D2EHPA-amine mixtures at different mole fractions of the amine in (**a**), (**b**) and (**c**), i.e., before, after and at the maximum of viscosity observed reported in Figure 1. Organic to aqueous phase volume ratio O/A = 1; initial concentration of cobalt = 0.008 mol·L^−1^.

Figure 5 shows extraction efficiencies of cobalt(II) from acidic chloride solutions (chloride concentrations ranging from 0.1 to 5 mol·L^−1^) by D2EHPA mixed with TOA, TIOA or TEHA) at different mole fractions of the amine. Mole fractions of the amine have been chosen in order to evaluate the influence of the speciation in the organic phase on cobalt(II) extraction. In particular, three regions have been studied: (i) mole fraction of the amine lower than the mole fraction corresponding to the maximum of viscosity as free D2EHPA is predominant, (ii) mole fraction of the amine equal to the maximum of viscosity as {R_3_NH^+^, L^−^}(HL)_a−1_ is the main species, and (iii) mole fraction of the amine greater than the mole fraction corresponding to the maximum of viscosity as the free amine is the predominant species in this region (see Figure 1 and Figure 4).

At a mole fraction of the amine lower than the maximum of viscosity, no cobalt(II) extraction occurs for chloride concentration lower than 5 mol·L^−1^ whatever the nature of the amine mixed with D2EHPA. At 5 mol·L^−1^ of chloride, only the mixtures containing TOA and TEHA extract cobalt(II) but the extraction efficiency remains very low. Cobalt(II) is likely not extracted by TOA or TEHA)-D2EHPA mixtures at x(amine) = 0.2 because there is no free amine (Figure 3d,e). Conversely, cobalt(II) may be partially extracted by {R_3_NH^+^, L^−^}(HL)_a−1_ via an anion extraction equilibria (a = 2 for TOA and a = 3 for TEHA).

At mole fraction of the amine corresponding to the maximum of viscosity in Figure 1, only {R_3_NH^+^, L^−^}(HL)_a−1_ species are present in solution. Figure 5c shows that cobalt(II) extraction is greatly increased. It confirms that {R_3_NH^+^, L^−^}(HL)_a−1_ can efficiently extract cobalt(II). However, extraction efficiency depends on both chloride concentration and the nature of the amine. The higher the chloride concentration, the higher the cobalt extraction. This is not surprising since the extraction involves an anion exchange equilibria and cobalt mainly exists as anions at high chloride concentration (CoCl_3_^−^ and CoCl_4_^2−^) [22,23]. Figure 5c shows that extraction performance increases in the following order: TEHA < TIOA < TOA. However, it is interesting to point out that cobalt(II) extraction by TIOA and TOA tends to be similar at chloride concentration greater than 3 mol·L^−1^. This is in accordance with the physicochemical part of this paper, which showed that branching of an amine seems to destabilize electrostatic interactions in solution. Therefore, the low extraction efficiency in D2EHPA-amine mixtures containing branched amine might result from low stability of {R_3_NH^+^, L^−^}(HL)_a−1_ species. 

Figure 5c confirms that an increase in mole fraction of the amine is responsible for an increase in cobalt(II) extraction efficiency. Figure 4d–f shows {R_3_NH^+^, L^−^}(HL)_a−1_ concentration decreases at the extent of an increase in the free amine concentration. Therefore, cobalt(II) extraction may involve two simultaneous extraction equilibria involving {R_3_NH^+^, L^−^}(HL)_a−1_ and the free amine when amine mole fraction is greater than the mole fraction corresponding to the maximum of viscosity. The presence of two species exhibiting extractive properties towards cobalt(II) is responsible for better cobalt(II) extraction efficiency.

**Figure 6:**
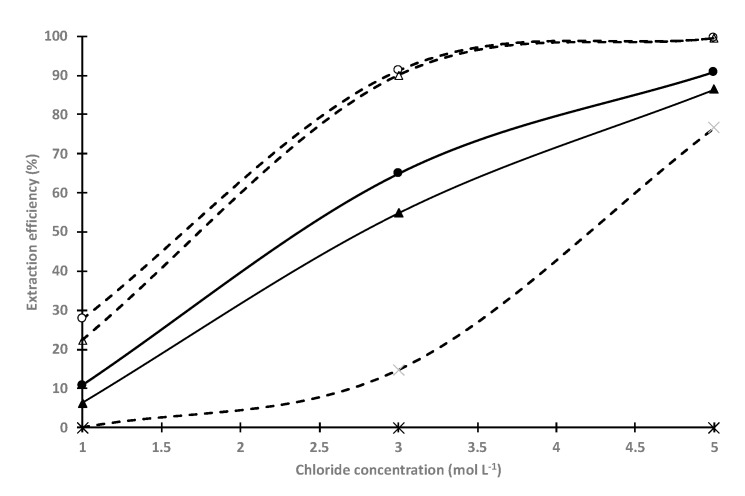
Comparison of cobalt extraction and nickel extraction efficiencies vs. initial chloride concentration by the D2EHPA-amine mixtures at different mole fractions of the amine (before, after and at the maximum of viscosity observed in Figure 1). White/doted-line: cobalt extraction; black/straight line: nickel extraction; circle: TOA-D2EHPA; triangle: TIOA-D2EHPA; cross: TEHA-D2EHPA. O/A = 1; initial concentration of cobalt and nickel = 0.008 mol·L^−1^.

The cobalt(II)-nickel(II) selectivity has been also investigated with D2EHPA-amine mixtures at x(amine) = 0.6 as a function of chloride concentration (Figure 6). For all mixtures, the selectivity decreases when chloride concentration increases. Similar extraction behaviour is observed between the D2EHPA-TOA and D2EHPA-TIOA mixtures and these systems exhibit low selectivity. The highest selectivity is obtained for D2EHPA-TEHA mixture as cobalt(II) extraction efficiency is equal to 77% at a chloride concentration of 3 mol·L^−1^ while there is no significant nickel(II) extraction. 

## 4. Conclusions

Viscosity, density and enthalpy have been determined at 25 °C in order to bring information about interactions and speciation in mixtures of organophosphorus acid (D2EHPA and Cyanex 272) and amine (TEHA, TIOA, TOA) as well as the effect of amine on the structuration/destructuration of the ionic liquids. As expected, this study shows interactions between D2EHPA and the amine are greater than between Cyanex 272 and the amine in accordance with the highest acidity of D2EHPA. This results likely in higher stability of D2EHPA-amine ionic liquids than Cyanex 272-amine ionic liquids. This results in higher viscosity of the D2EHPA-amine mixtures than the Cyanex 272 systems. However, the magnitude of the difference between the experimental molar volume and the ideal molar volume in absence of interaction and reaction is almost the same whatever the nature of organophosphorus acid or amine. This means that the addition of the amine in the organophosphorus acid does not significantly organize the solution in spite of the formation of the strong interactions. Conversely, it appears that branching of the amine destabilizes electrostatic interactions in solution. A simple model describing the variation of the viscosity as a function of amine mole fraction shows the presence of free amine, free D2EHPA or free Cyanex 272 and {R_3_NH^+^, L^−^}(HL)_a−1_ in organophosphorus compound-amine mixtures at various concentration depending on the mole fraction of the amine.

Extraction tests of D2EHPA-amine mixtures at various mole fraction of the amine show that {R_3_NH^+^, L^−^}(HL)_a−1_ can extract cobalt(II). The extraction efficiency depends on the percentage of the free amine and {R_3_NH^+^, L^−^}(HL)_a−1_ present in solution. The highest selectivity is obtained with D2EHPA-TEHA mixture (x(amine) = 0.6) as cobalt(II) extraction efficiency is equal to 77% at a chloride concentration of 3 mol·L^−1^ while there is no significant nickel(II) extraction.

## Figures and Tables

**Table 1:** Viscosities and densities for D2EHPA-amine mixtures at 25 °C (uncertainties on mole fraction = ±0.1%, dynamic viscosity = ±2%, density = ±0.2%).

	D2EHPA-TEHA		D2EHPA-TIOA		D2EHPA-TOA	
x (amine)	η (cP)	ρ (g·L^−1^)	η (cP)	ρ (g·L^−1^)	η (cP)	ρ (g·L^−1^)
0.00	36.3	0.972	36.3	0.972	36.1	0.972
0.13	324.7	0.960	172.9	0.956	147.3	0.955
0.14	395.1	0.958	202.2	0.955	176.9	0.953
0.17	502.9	0.956	226.7	0.954	220.7	0.950
0.19	633.9	0.953	304.8	0.950	325.2	0.945
0.25	934.0	0.944	686.7	0.940	470.4	0.938
0.33	788.4	0.930	1305.1	0.930	744.5	0.927
0.39	561.5	0.918	1679.8	0.923	824.1	0.917
0.48	335.7	0.900	1027.9	0.901	420.3	0.898
0.58	204.2	0.882	402.9	0.886	163.5	0.878
0.79	61.7	0.847	71.2	0.848	31.4	0.837
1.00	17.5	0.815	11.0	0.813	8.3	0.808

**Table 2:** Viscosities and densities for Cyanex 272-amine mixtures at 25 °C (uncertainties on mole fraction = ±0.1%, dynamic viscosity = ±2%, density = ±0.2%).

	Cyanex-TEHA		Cyanex-TIOA		Cyanex-TOA	
x (amine)	η (cP)	ρ (g·L^−1^)	η (cP)	ρ (g·L^−1^)	η (cP)	ρ (g·L^−1^)
0.00	124.2	0.815	124.9	0.813	124.6	0.808
0.13	83.8	0.832	400.0	0.834	325.0	0.829
0.15	80.3	0.850	480.0	0.853	353.2	0.850
0.18	75.6	0.859	537.0	0.866	390.4	0.862
0.20	70.3	0.867	562.5	0.874	404.7	0.875
0.24	65.8	0.875	489.9	0.883	340.1	0.881
0.27	56.5	0.885	435.8	0.895	299.7	0.893
0.36	46.6	0.891	227.4	0.897	149.9	0.896
0.43	39.7	0.894	116.6	0.901	89.9	0.901
0.51	34.4	0.897	73.2	0.904	43.2	0.902
0.61	29.4	0.900	41.4	0.906	26.4	0.905
0.80	22.1	0.901	20.4	0.909	13.5	0.906
1.00	17.7	0.916	10.9	0.916	8.3	0.916

**Table 3:** Global enthalpy for D2EHPA- and Cyanex 272-amine mixtures at 25 °C (uncertainties on mole fraction = ±0.1%, enthalpies = ±3%).

D2EHPA						Cyanex 272			
TEHA		TIOA		TOA		TIOA		TOA	
x(amine)	H (J·mol^−1^)	x(amine)	H (J·mol^−1^)	x(amine)	H (J·mol^−1^)	x(amine)	H (J·mol^−1^)	x(amine)	H (J·mol^−1^)
0.11	−4331.0	0.20	−10,498.8	0.10	−6780.1	0.10	−2794.0	0.10	−2824.5
0.23	−7605.3	0.30	−12,514.6	0.21	−13,137.3	0.20	−5611.7	0.20	−6640.6
0.41	−6831.8	0.38	−14,455.3	0.31	−17,921.9	0.30	−6640.2	0.30	−7346.5
0.50	−6375.6	0.49	−14,446.2	0.40	−17,900.4	0.39	−6342.3	0.38	−7132.9
0.56	−5950.2	0.59	−13,809.4	0.57	−14,032.3	0.50	−6006.0	0.50	−5794.3
0.66	−4811.6	0.67	−12,617.9	0.71	−10,513.4	0.60	−5513.4	0.59	−4888.8
0.75	−3993.5	0.78	−8095.2	0.90	−3609.2	0.70	−4110.0	0.69	−3814.1
0.86	−1828.6	0.89	−4299.0			0.80	−3027.1	0.82	−2147.2
						0.90	−1802.3	0.90	−1349.5

**Table 4:** Redlich-Kister parameters of *V_m_*^≠^ at 25 °C for the D2EHPA-amine and Cyanex 272-amine mixtures.

	D2EHPA			Cyanex 272		
	TOA	TIOA	TEHA	TOA	TIOA	TEHA
A	−28.164	0	0	31.791	7.294	0
B	3.242	−12.909	−30.578	3.910	4.519	0.832
C	47.528	11.669	−12.034	−22.358	−12.723	−0.362
D	18.944	15.831	33.124	12.240	12.272	9.594
E	−32.904	−31.707	−23.300	−17.542	−17.657	−5.050

**Table 5:** Stoichiometry of the amine-organophosphorus species in ionic liquids.

	D2EHPA	Cyanex 272
TOA	a = 2 R_3_NHL(HL) η_IP_ = 900 cP	a = 4 R_3_NHL(HL)_3_ η_IP_ = 440 cP
TIOA	a = 2 R_3_NHL(HL) η_IP_=1900 cP	a = 4 R_3_NHL(HL)_3_ η_IP_ = 600 cP
TEHA	a = 3 R_3_NHL(HL)_2_ η_IP_ = 1100 cP	_
